# Differential effects of four intramuscular sedatives on cardiorespiratory stability in juvenile guinea pigs (*Cavia porcellus*)

**DOI:** 10.1371/journal.pone.0259559

**Published:** 2021-11-15

**Authors:** Ryan P. Sixtus, Cholawat Pacharinsak, Clint L. Gray, Mary J. Berry, Rebecca M. Dyson

**Affiliations:** 1 Department of Paediatrics and Child Health, & Centre for Translational Research, University of Otago, Wellington, New Zealand; 2 Department of Comparative Medicine, Stanford University, Stanford, CA, United States of America; Belgrade University Faculty of Medicine, SERBIA

## Abstract

**Background:**

Non-invasive physiological monitoring can induce stress in laboratory animals. Sedation reduces the level of restraint required, thereby improving the validity of physiological signals measured. However, sedatives may alter physiological equilibrium introducing unintended bias and/or, masking the experimental outcomes of interest. We aimed to investigate the cardiorespiratory effects of four short-acting sedatives in juvenile guinea pigs.

**Method:**

12 healthy, 38 (26–46) day-old Dunkin Hartley guinea pigs were included in this blinded, randomised, crossover design study. Animals were sedated by intramuscular injection using pre-established minimum effective doses of either alfaxalone (5 mg/kg), diazepam (5 mg/kg), ketamine (30 mg/kg), or midazolam (2 mg/kg) administered in random order with a minimum washout period of 48 hours between agents. Sedative depth, a composite score comprised of five assessment criteria, was observed every 5-min from dosing until arousal. Physiological monitoring of cardiorespiratory status included measures of heart rate, blood pressure, respiratory rate, and peripheral microvascular perfusion.

**Results:**

Ketamine and alfaxalone were most effective in inducing stable sedation suitable for physiological monitoring, and diazepam less-so. Midazolam was unsuitable due to excessive hypersensitivity. All sedatives significantly increased heart rate above non-sedated control rates (*P*<0.0001), without altering blood pressure or microvascular perfusion. Alfaxalone and ketamine reduced respiratory rate relative to their control condition (*P*<0.0001, *P* = 0.05, respectively), but within normative ranges.

**Conclusion:**

Ketamine and alfaxalone are the most effective sedatives for inducing short duration, stable sedation with minimal cardiorespiratory depression in guinea pigs, while diazepam is less-so. However, alfaxalone is the most appropriate sedative for longitudinal studies requiring multiple physiological timepoints.

## Introduction

Many routine physiological assessments in laboratory animals require some level of restraint, either chemical or mechanical, to enable data acquisition, however, neither option can be considered optimal. Mechanical restraint may induce significant physiological stress, impairing the quality and validity of the assessments to be performed, whereas chemical restraint, either inhalational or injectable anaesthesia, may be confounded by complexities associated with the route of administration (e.g., vascular access in guinea pigs) [1, 2]. Additionally, the side-effects of anaesthesia include cardiovascular and respiratory depression, thereby limiting its use for a range of physiological studies [2–4]. Short-acting sedative agents may therefore offer a compromise, mitigating the confounding effects of manual restraint or full anaesthesia without abolishing key cardio-respiratory outcomes of interest.

Despite their wide utility, the cardiorespiratory impact of short acting sedatives in laboratory species remains largely unresolved [5]. The favoured sedatives change depending on procedures being performed, depth of sedation required, and the conventions of specific laboratories. Commonly used sedatives act on a range of different pathways, their use determined by the strength of on- and off-target effects. Agents such as alfaxalone and benzodiazepines act on GABAergic pathways to depress cortical function [6], whereas ketamine and nitrous oxide act on N-methyl-D-aspartate (NMDA) glutamatergic pathways to inhibit cortical excitability [7]. Alfaxalone has been frequently used as an induction agent, and recently has been used to achieve sedation for short duration non-noxious procedures while maintaining stable core body temperature and heart rate (HR) [8]. Benzodiazepines like midazolam and diazepam, are often combined with opioids or alpha-2 agonists (e.g., Medetomidine-Midazolam-Fentanyl (MMF)) to offset their lack of analgesic properties [6]. Benzodiazepines, however, also produce off-target vasodilation, which alters cardiovascular function [6, 8], limiting their usefulness in cardiovascular studies. Ketamine is effective in producing dissociative sedation and effective analgesia with limited cardiorespiratory depression but is also often paired with an alpha-2 agonist (e.g., medetomidine) to counter the effects of ketamine on muscle tone and movement [6]. The effects induced by these various sedatives are often short in duration (<1h) and limited in their sedative depth, such that only short duration, minimally invasive procedures can be performed.

Guinea pigs (*Cavia porcellus*) are a common laboratory species, used for a wide variety of research modalities [2]. They are, however, notoriously difficult to safely and effectively anaesthetise [1]; and this is also true for sedation. A lack of easily accessible venous access requires that injectable agents are safe for subcutaneous (SC) or intramuscular (IM) administration. IM administration of ketamine, for example, can produce skin irritation and tissue necrosis around the injection site [9], frequently resulting in self-mutilation following sedation [10]. Furthermore, once sedated, physiological responses in guinea pigs are notoriously unpredictable [11]. This can confound comparisons of physiological effects between species, and requires species-specific investigations to characterise the physiological effects of sedative agents [8]. Finally, there is limited research to offer an ‘ideal’ sedative agent that does not have associated negative cardiorespiratory effects. We have therefore sought to characterise a) the cardiorespiratory effects, and b) the sedative stability, of four different commonly used sedative agents in guinea pigs.

## Material and methods

### Sedatives

Alfaxalone (10 mg.mL^-1^, Alfaxan Multidose, Jurox, UK), diazepam (5 mg.mL^-1^, Ilium diazepam, Troy Laboratories, Australia), ketamine (100 mg.mL^-1^, Phoenix Pharm, NZ), midazolam (5 mg.mL^-1^, Hypnovel, Roche Products, NZ).

### Animals

All procedures were approved by the University of Otago, Wellington Animal Ethics Committee, and conformed to Health Research Council of New Zealand code of practice for the care and use of animals for scientific purposes, and are reported according to the ARRIVE guidelines [12]. Outbred, SPF Dunkin Hartley guinea pigs were sourced from the Biomedical Research Unit, University of Otago Wellington. To prevent litter bias, no more than 2 pups/sex/litter were used in any arm of the study. Guinea pigs were housed individually in a 12:12hr light- and temperature-controlled environment, with *ad libitum* access to standard guinea pig chow (Specialty Feeds, Glen Forrest, Australia) and vitamin C-enriched water (1 g.L^-1^).

#### Study I–sedative trial

Twelve age-matched adolescent Dunkin Hartley guinea pigs (male (n = 6) and female (n = 6); mean age: 38d, (26-46d); mean weight: 364g (287-428g)) were included in this randomised crossover design study [13]. Sequence of sedation treatments was allocated using a computerised method (RAND function in Microsoft Excel) at commencement of study [14].

#### Study II–normative blood pressure

Twenty age-matched animals (male (n = 10) and female (n = 10); mean age: 38d (34-41d); mean weight: 362g (314–423)) were included for the purpose of establishing normalised systolic blood pressure (SBP) ranges in non-sedated juvenile Dunkin Hartley guinea pigs. Animals of corresponding age to Study I were randomly selected from the colony. Due to movement associated with NIBP cuff inflation, recordings were not available for n = 3 males and n = 3 females (due to excess movement and subsequent inability to obtain clear pulse and occlusion recordings). Thus, normative data is based only on those animals with a completed NIBP trial (male (n = 7) and female (n = 7); mean age: 38d (34-41d); mean weight: 356g (314–423)).

### Procedure

#### Study I–sedative trial

Animals underwent five cardiovascular assessments, one under each sedative, and one conscious control (measures performed in non-sedated animals; no vehicle injection given) in random order. Sedatives assessed included alfaxalone (5mg.kg^-1^), diazepam (5mg.kg^-1^), ketamine (30mg.kg^-1^), and midazolam (2mg.kg^-1^). Dosage was guided by published reports [15], and veterinary practice (co-author CP; and Institutional Veterinarian–University of Otago). Where a range of dosages was reported, a dose-optimisation study was performed (data not presented). Each trial was completed at the same time of day to minimise circadian bias. At commencement of each trial, animals were assigned an American Society of Anaesthesiologists Veterinarian Technicians Score (ASA-VTS) based on physical examination (ASA I: minimal risk–ASA V: extreme risk) [8], and baseline measures of sedation/activity were recorded according to five parameters: movement, body tone, reaction to manipulation, posture, and righting reflex (Table 1). These five assessments were summed in an unweighted manner to provide an overall cumulative sedative depth, with a range of 0–19, where “0” represents normal guinea pig behaviour, and higher scores reflecting greater sedative depth, “19” reflecting excess sedation. In addition to these parameters, observations outside of these parameters were noted under the categories of ambulatory, chewing, hyperactive, change in respiration, squinting, shivering, twitching, uncoordinated, and vocalisation as more subjective markers of behaviour outside of the formal monitoring matrix in order to fully capture animal’s state during the trial.

**Table 1:** Five monitored criteria contributing to cumulative sedative depth score.

Score	Movement	Body Tone	Reaction to Manipulation	Posture	Righting Reflex
**0**	Normal coordinated movement	Rigid, hunched, waking tone	Normal reaction	Normal	Regained sternal recumbency immediately
**1**	Coordinated movement	Relaxed but upright, muscle tone present	Decreased response (flinch/ movement/ noise)	Head up but sitting	Regained sternal recumbency within 5-10s
**2**	Uncoordinated movement	Drowsy, recumbency, floppy	Minimal response (slight flinch/ movement)	Head down sternal recumbency	Attempted to regain sternal recumbency but failed
**3**	Infrequent uncoordinated movement	Sedate, Atonal	No response	Lateral recumbency	Did not attempt to reposition
**4**	Infrequent weak uncoordinated movement			Dorsal recumbency but responsive to stimuli	
**5**	No movement			Dorsal recumbency and unresponsive	

Total score of 0 corresponding to an awake, alert and coordinated animal; a total score of 19 corresponding to a deeply sedated, atonal and unresponsive animal.

After baseline assessments of activity, animals were lightly restrained and sedative dose administered, before being placed in a quilt-lined cage for sedatives to take effect with minimal tactile, auditory or visual stimulation. All sedatives were provided by IM injection in the hindlimb(s). Where drug volume exceeded 0.3 ml.kg^-1^ (diazepam, midazolam), doses were split between both legs [15] Injection sites were additionally alternated between trials to minimise potential discomfort from prior injections. Sedative depth was assessed in 5 min intervals from injection until completion of sedation. Sedation duration was calculated from observed onset of sedation through to arousal of the animal, with the majority of monitored signs returning to “normal” (Table 1). At onset of sedation, animals were transferred to a heated surface inside a temperature- and humidity-controlled chamber (25°C; 30%RH) for cardiovascular assessment.

Cardiovascular assessment consisted of electrocardiography (needle electrode, ADInstruments, Dunedin, New Zealand) positioned over the inferior sternum and interscapular sites (earth: right rump) [16], SBP (non-invasive blood pressure, rat tail cuff, ADInstruments), positioned over right forelimb, and microvascular perfusion measured over proximal (interscapular) and distal (dorsal ear) skin sites using laser Doppler flowmetry (LDF; Probe 457, PeriFlux 5001, Perimed, Jarfalla, Sweden) affixed to depillated skin sites using modified TCO_2_ fixation rings and double sided tape [17]. Respiratory rate was additionally measured using a pulse transducer (ADInstruments) positioned on the anterior abdominal wall, below the level of the diaphragm. Throughout the trials, animals were minimally restrained as required, to avoid excess tactile stimulation by assessors.

**Fig 1:**
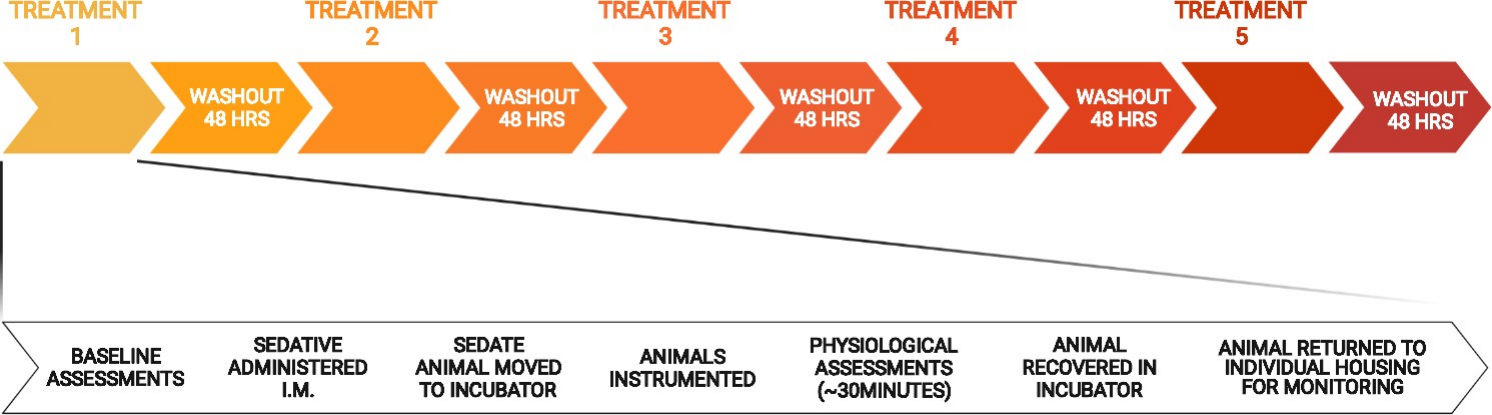
Study I experimental timeline. Animals underwent five cardiovascular assessments, one under each sedative, and one conscious control (measures performed in non-sedated animals; no vehicle injection given) in random order with a 48hr washout between each treatment (top panel). During each assessment (bottom panel) animals had baseline assessments of activity (Table 1), received sedative, and then were instrumented and underwent cardiorespiratory recordings (electrocardiogram, non-invasive blood pressure, laser Doppler perfusion monitoring, respiratory rate assessment). Animals were allowed to recover on a heat pad within a heat-controlled incubator, before being returned to individual housing for post-intervention monitoring. Created with BioRender.com.

Animals were recovered for 48hr between trials to allow complete drug washout and recovery between trials. Upon recovery form each trial, animals received a body weight measurement, alongside a measure of wellbeing (B.A.R. [bright, alert, responsive] score and general clinical signs [inactive, hunched posture, coat rough, inflammed depilation sites, irritation of injection sites, and dehydration; normal = 0, and 1, 2, 3 for increasing severity]). An overview of the study design is presented in Fig 1.

#### Study II–normative blood pressure

Animals were randomly selected from the colony for one session of NIBP monitoring to establish normative SBP outside of Study I population and in the absence of additional physiological measures. Animals were relocated to the experimental room and placed in a quilt lined box to acclimate to the new surroundings for 20 min. They were then placed on a heated surface and lightly restrained as per *Study I*. An NIBP cuff was positioned over the right forelimb and, following a settling period of 30 min, inflated in 5 min intervals until three artefact-free recordings of SBP were obtained or 60 min had elapsed. Normative blood pressure recordings were assessed as per *Study I*.

### Data analysis

ECG, NIBP, and respiration were sampled using PowerLab (ADInstruments) at a rate of 1 k.s^-1^, with an analogue notch filter applied, and recorded in LabChart (ADInstruments). HR and respiration were derived using peak-to-peak analysis. LDF collected alongside central cardiovascular assessments was recorded at 32 Hz with a time constant of 0.03 s, and assessed in arbitrary perfusion units (PU; equipment calibrated prior to each experimental day) and analysed using custom software (PSW2; Perimed). In order to limit confounding effects of probe temperature on the cutaneous vascular response to sedatives, LDF measures were taken without controlling for probe temperature, according to established protocols [17–19].

Due to logistical constraints (e.g., level of consciousness) in blinding the experimenter (RPS) to all conditions, the experimenter was blinded to the identity of the sedative agent used both at the time of administration and during data analysis. The clinical condition of the animals was verified by a second experienced assessor (RMD), also blinded to experimental group at time of analysis. Whilst recorded continuously, cardiovascular, respiratory and microvascular perfusion measures were assessed across three separate epochs during sedation, with a 60s window of representative, artefact-free data selected for further analysis.

### Statistical analyses

Normality was assessed and confirmed using the Kolmogorov-Smirnov normality test. GraphPad Prism 7.04 (GraphPad Software, California, USA) was used for statistical analysis and generation of figures. Data were analysed using repeated measures mixed-effects or two-way ANOVA, with time or sedative treatment as the main factors. When compared to control, cumulative sedative depth scores were truncated to key timepoints (‘baseline’, ‘removed from cage’, ‘physiological measures 1’ (P1), physiological measures 2’ (P2), ‘back to cage’, and ‘end’) to ensure comparison across the different durations of each sedative. Results were deemed statistically significant at *P*≤0.05 and reported as ±95% CI unless otherwise stated.

### Data availability

All relevant data associated with sedative depth and cardiorespiratory findings are included in the paper and/ or its supplementary information files. S1–S5 Tables provide the individual data for each monitored sign for sedative depth data. The raw data files (recordings) supporting the cardiorespiratory findings of this study are available from the authors on reasonable request.

## Results

### Study I: Animal and sedative characteristics

#### Animal characteristics

Twelve animals completed the randomized sedation trial (*Study I*). The combined physical characteristics are presented in Table 2. Animal weight at each treatment were not different from control (*P* = 0.45). 24hr weight change (%) was not different between treatments (Table 2), with all animals, on average, gaining weight over the first 24 hours after sedation across all treatments. The mean age at study start in Study I was 33d with a standard deviation of ±4 days. Average age (±S.D.) for each treatment was not different from control, or between treatments (*P =* 0.85; control 37±6d, alfaxalone 38±4d, ketamine 37±5d, midazolam 36±5d, diazepam 38±4d). Of the 20 animals selected for the normalized blood pressure trial (*Study II*), useable data characterising non-invasive measures of SBP in a non-sedated animal habituated to handling were acquired in fourteen.

**Table 2:** Animal characteristics for alfaxalone, diazepam, ketamine and midazolam.

	Male	Female	24hr weight change (%) (n = 12)	24hr BAR score (n = 12)
	Weight (g) (n = 6)	Weight (g) (n = 6)		
Control	362.5±47.0	360.0±43.6	1.36±1.23	0
Alfaxalone	367.0±44.9	340.8±36.8	1.23±3.88	0
Diazepam	375.2±41.9	360.5±37.8	1.14±3.78	0
Ketamine	371.3±59.9	359.0±42.6	1.04±3.82	0
Midazolam	365.8±36.1	365.2±38.9	1.09±2.92	0
Normative Control	341.0±67.8	355.3±23.1	N/A	N/A

24hr weight change is displayed as mean±S.D.

Both diazepam (3 instances) and ketamine (1 instance) were associated with instances irritation of the injection site. This presented as dragging of the hind limb following arousal from sedation, and scratching and chewing of the affected limb. This did not appear to be associated with tenderness of the site and there was no obvious swelling of the limb. In most instances, this resolved within 24 hours of administration (1 animal had persistent irritation (~4 days) associated with diazepam injection). This is in line with earlier reports that IM ketamine and diazepam may drive tissue irritation and even necrosis at the injection site. No necrosis was observed at injection sites following any of the sedatives, however animals were not observed long-term beyond the study period to capture this data. Doppler/ECG monitoring sites showed mild irritation (dry, red skin) in some animals, likely from repeated shaving/depilation, however this happened in relatively few animals and did not appear to effect signal acquisition.

#### Sedative characteristics over time

**Fig 2:**
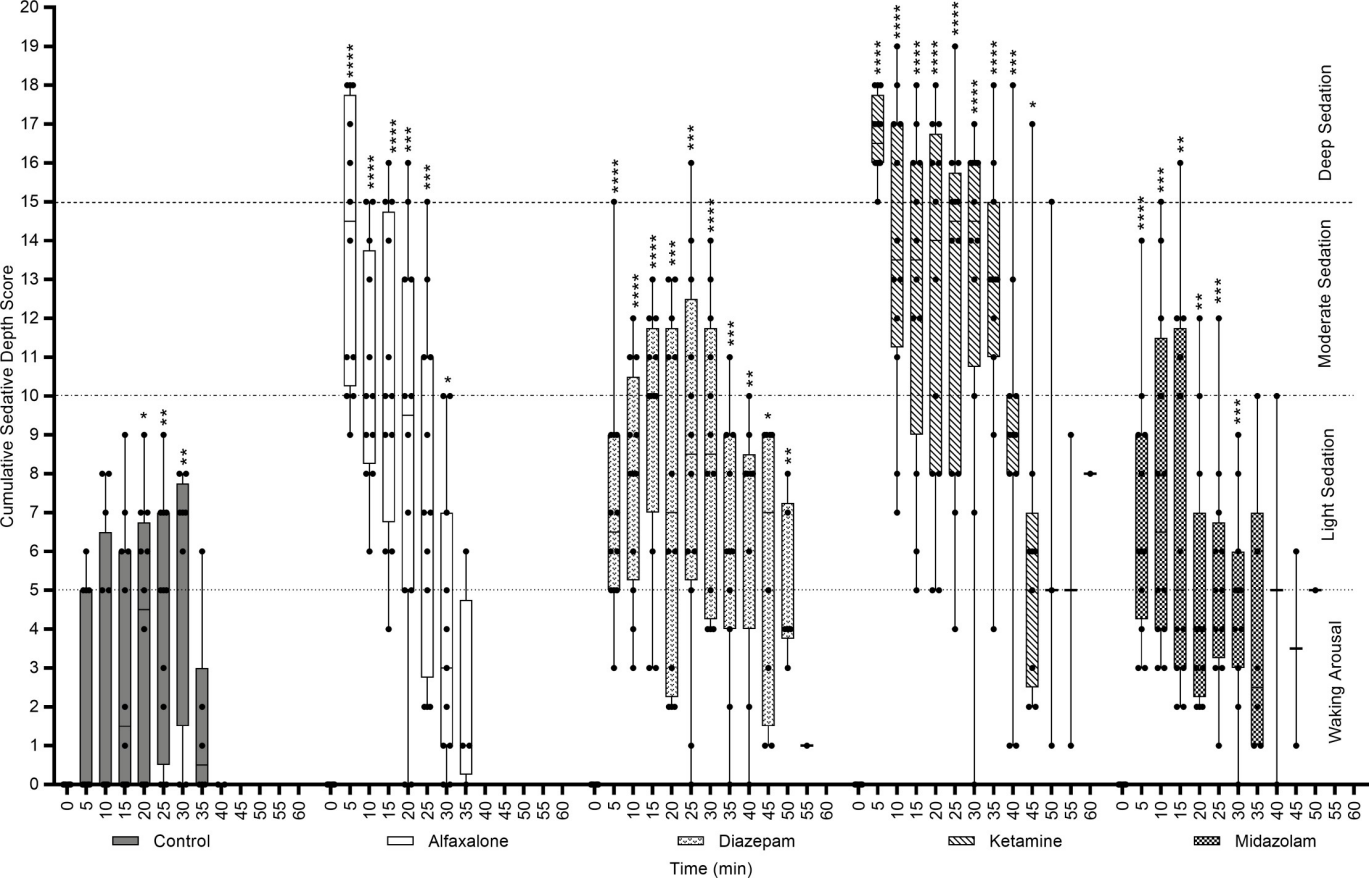
Cumulative sedative depth across the course of physiological monitoring for each condition: Control, alfaxalone, diazepam, ketamine, midazolam. Accumulated score of five monitored signs: movement (0–5), body tone (0–3), reaction to manipulation (0–3), posture (0–5), righting reflex (0–3); ranging from 0–19. Horizontal dashed lines at a sedative depth of 5, 10, and 15, indicate zones of sedative depth, whereby 0–5 animals presented waking arousal, 5–10 animals were mildly sedated, 10–15 animals were moderately sedated, and 15–19 animals were highly sedated. At time 0, all animals scored 0 (“normal”) across all parameters. No weighting has been applied to the individual signs that contribute to the cumulative depth score, therefore it is possible that the score for each sedative may be skewed by excess change in individual signs (e.g., excess body tone under ketamine sedation). S1–S5 Tables provide the individual data for each monitored sign, as well as number of animals monitored at each time point (all studies started with n = 12, but due to length of sedative duration, there is differential drop out between sedatives). *** significance in relation to baseline (0 min).

All drugs produced significant changes in the cumulative sedative depth score in comparison to pre-sedation baseline (Fig 2; contributing measures individually reported in S1–S5 Tables). The most rapid sedation occurred under alfaxalone (5.16±0.32 min), with ketamine (6.16±1.05 min) and midazolam (6.44±1.39 min) also producing rapid sedation, whereas onset of sedation under diazepam was significantly longer than other sedatives (12.25±1.46 min; *P* = 0.0006). Sedation duration was similar between alfaxalone, diazepam and midazolam (27.14±1.52 min, 33.00±3.31 min and 28.15±3.41 min, respectively), with ketamine lasting significantly longer than both alfaxalone and midazolam (41.08±5.02 min; *P* = 0.0013; *P* = 0.016), but not diazepam (*P* = 0.15). Peak effects for ketamine and alfaxalone occurred with onset (comparison to baseline: ~5 min, *P*<0.0001 for both), whereas peak effects occurred around 10- and 15-min for midazolam and diazepam, respectively (*P*<0.0001 and *P* = 0.0005, respectively). Ketamine produced the deepest sedation, closely followed by alfaxalone (16.7±0.6 and 13.9±2.2 out of 19, respectively; *P*<0.0001). Diazepam and midazolam produced shallower sedation (9.3±2.1 and 7.6±2.7). In the absence of drugs, control animals also demonstrated a significant calming effect after 20 min of light restraint (20 min: 3.7±2.2, *P* = 0.019; 25 min: 4.2±2.0, *P* = 0.0041; 30 min: 5.4±2.8, *P* = 0.0083).

#### Sedative characteristics in comparison to control

All drugs produced significant changes in the cumulative sedative depth score in comparison to the control condition (Table 3). Baseline sedative scores were not different from control. Sedative depth was adequately achieved in all sedative treatments prior to removing the animal from the cage, as assessed using the sedative depth score, with ketamine and alfaxalone producing the greatest immediate effects (ketamine, 5 min: 16.8±0.7, *P*<0.0001; alfaxalone, 5 min: 13.9±2.3, *P*<0.0001), followed by diazepam (10 min: 7.8±2.0, *P* = 0.0007), and midazolam (6 min: 7.1±2.0, *P* = 0.0038). Sedation under ketamine and alfaxalone remained highly significant from control throughout physiological monitoring (P1: ketamine, 12 min:13.0±2.4, *P*<0.0001; alfaxalone, 11 min: 10.6±1.8, *P* = 0.0002; P2: ketamine, 19 min: 13.5±2.6, *P*<0.0001; alfaxalone, 16 min: 10.9±2.6, *P* = 0.0005); and animals under ketamine remained highly sedated upon returning to cage (28 min: 12.7±2.4, *P*<0.0001), whereas sedation under alfaxalone reduced more rapidly toward conscious levels (22 min: 8.6±2.7, *P* = 0.0059). Sedation was less effective under both diazepam and midazolam. By ‘P1’ sedative depth was only slighly higher than control when animals were removed from cage, becoming less different as the sedative score rose in control animals as they relaxed during monitoring (control, 11 min: 3.5±2.4; diazepam, 17 min: 9.0±2.6, *P* = 0.0086; midazolam, 11 min: 8.1±2.8, *P* = 0.04). Neither diazepam nor midazolam were significantly different from control by ‘P2’ (control, 18 min: 3.7±2.2; diazepam, 23 min: 7.1±3.2, *P* = 0.20; midazolam, 17 min: 6.8±3.0, *P* = 0.24), however, diazepam became significantly different from control when returned to cage, primarily due to a reduction in control sedative score (control, 26 min: 2.9±2.2; diazepam, 32 min: 7.3±2.1, *P* = 0.016). Animals sedated with alfaxalone, diazepam and midazolam all experienced hyperreactivity to experimental implementation. Under alfaxalone, animals settled within 5-min, whereas under diazepam and, in particular, midazolam were more variable, with five animals excessively hypersensitive to noise and touch under midazolam, which lead to physiological measures being abandoned in three animals.

**Table 3:** Standardised score of cumulative sedative depth.

Treatment		Removed from Cage	P1	P2	Returned to Cage	End
Control	Time	5.00 ± 0.00	10.50 ± 1.06	17.44 ± 2.02	25.5 ± 2.55	31.15 ± 2.59
	Score	2.3 ± 1.8	3.5 ± 2.4	3.7 ± 2.2	2.9 ± 2.2	2.3 ± 1.8
Alfaxalone	Time	5.00 ± 0.00	10.50 ± 1.57	15.50 ± 1.57	22.30 ± 2.37	31.40 ± 3.15
	Score	13.9 ± 2.3****	10.6 ± 1.8***	10.9 ± 2.6***	8.6 ± 2.7**	1.3 ± 0.9
Diazepam	Time	10.25 ± 0.49	17.30 ± 1.54	23.20 ± 2.12	32.30 ± 3.18	44.10 ± 3.58
	Score	7.8 ± 2.0***	9.0 ± 2.6**	7.1 ± 3.2	7.3 ± 2.1*	3.9 ± 1.8
Ketamine	Time	5.00 ± 0.00	12.30 ± 1.54	18.45 ± 1.46	28.20 ± 2.31	47.05 ± 5.35
	Score	16.8 ± 0.7****	13.0 ± 2.4****	13.5 ± 2.6****	12.7 ± 2.4****	3.4 ± 2.0
Midazolam	Time	5.50 ± 1.06	11.15 ± 1.17	17.05 ± 1.27	24.10 ± 1.38	32.55 ± 3.30
	Score	7.1 ± 2.0**	8.1 ± 2.8*	6.8 ± 3.0	5.5 ± 1.6	3.3 ± 1.1

All scores were 0 at Baseline. Time (min) started following injections, events standardised to 5 min intervals when scores of sedative depth were assessed. Key events during sedation included ‘removed from cage’ at which point animals were deemed sedated enough to begin physiological monitoring; ‘P1’ during which physiological measures were applied, ‘P2’, during late physiological monitoring; Returned to cage, when physiological measures were complete; and End whereupon animals were deemed to have recovered.

Statistics compared to control.

* *P* = 0.05,

** *P* = 0.001,

*** *P* = 0.0001,

**** *P*<0.0001.

### Cardiorespiratory physiology

#### Heart rate

**Fig 3:**
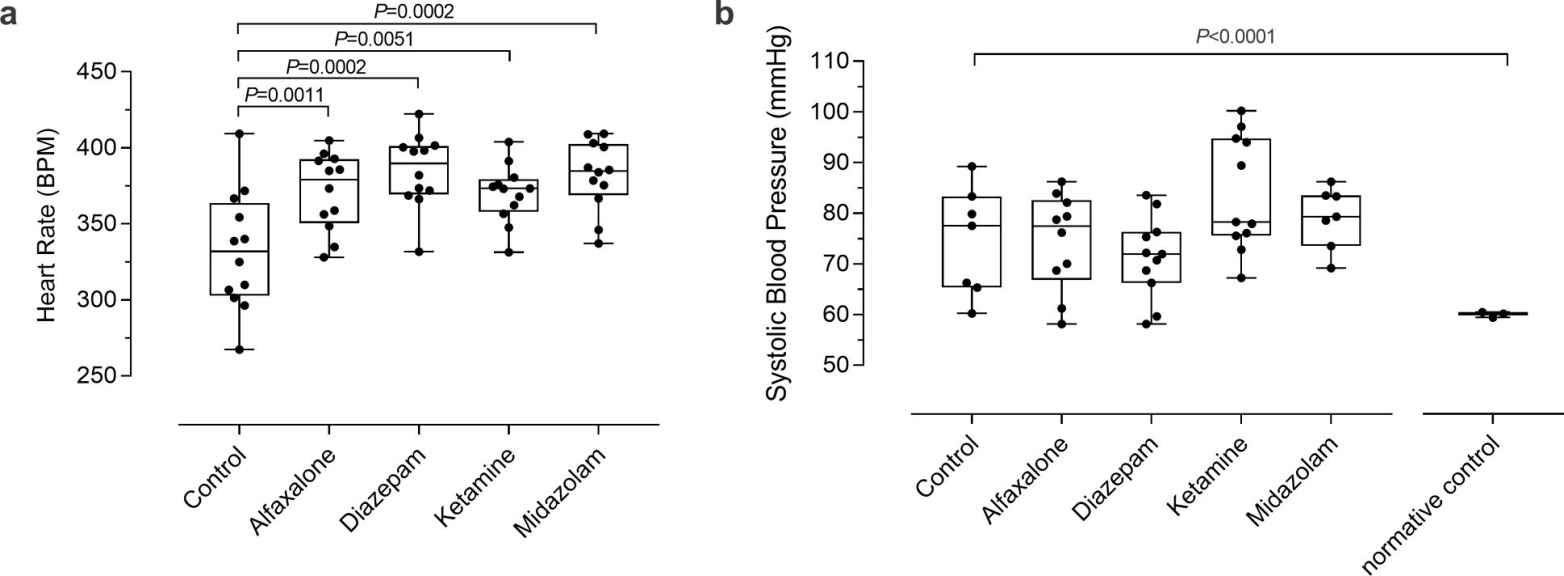
Heart rate (a) and systolic blood pressure (b) response to sedative agents. Normative control blood pressure from study II is displayed alongside study I. Significance indicates difference from control group (a), or from normative control (b). n = 12 for all groups in (a), however, due to noise/movement in blood pressure recordings, n for (b) was affected: control n = 7, alfaxalone n = 10, diazepam, ketamine and midazolam n = 11. For normative blood pressure, n = 14 (of 20).

HR was obtained for all animals during all experimental manipulations. HR of control animals during monitoring was 332±25 b.min^-1^, with a significant difference between males and females (male-female HR difference: 36 b.min^-1^; *P =* 0.04). This was the only sex difference across all measured physiological variables. Administration of all sedatives increased HR significantly above control levels (*P*<0.0001; Fig 3A). Ketamine eliciting the smallest increase in comparison to control (difference: 37.6 b.min^-1^, *P* = 0.005), followed by alfaxalone (39.1 b.min^-1^, *P* = 0.001), midazolam (49.6 b.min^-1^, *P* = 0.0002), and diazepam (52.8 b.min^-1^, *P* = 0.0002).

#### Systolic blood pressure

Control SBP was 74.5±9.9 mmHg. SBP did not significantly differ from control following treatment with any of the sedative agents tested (Fig 3B). Movement artifact in both control and sedated animals as a result of blood pressure cuff inflations meant that BP measures were not available for all animals. The number of animals with successful SBP measures was 7/12 (58%) control animals, 10/12 (83%) under alfaxalone, and 11/12 (92%) under each diazepam, ketamine, and midazolam.

#### Microvascular perfusion

**Fig 4:**
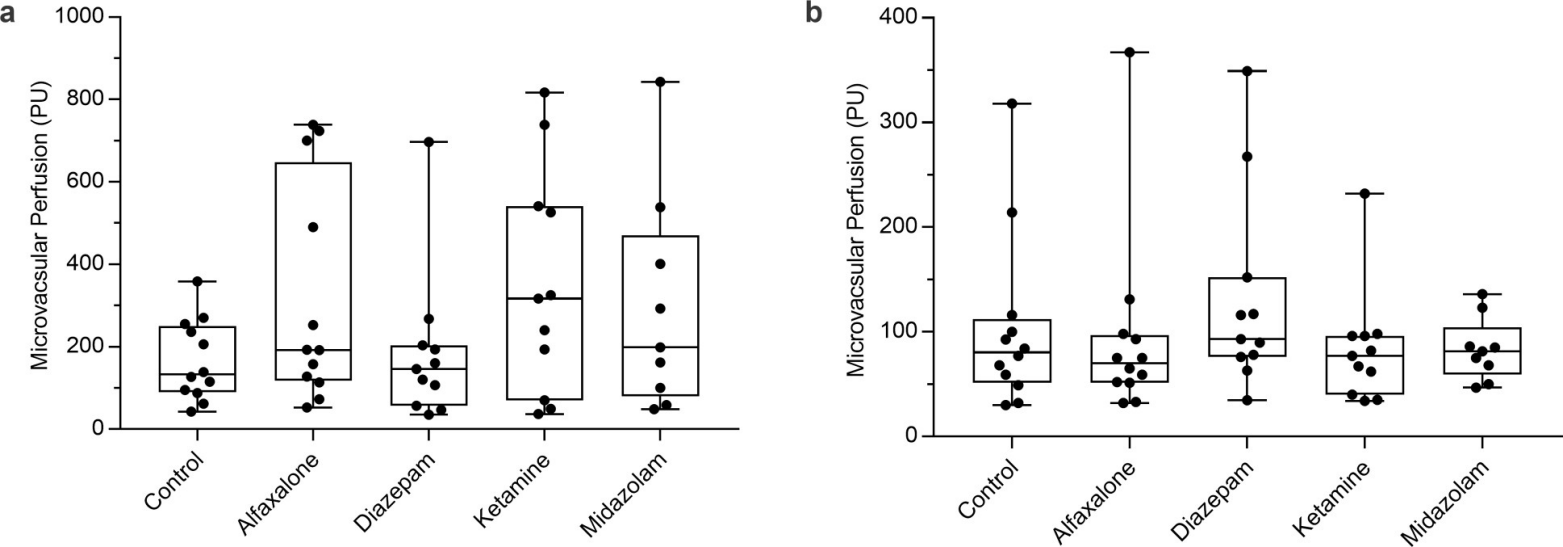
Distal (a) and proximal (b) microvascular perfusion response to different sedative agents. Due to noise/movement, n = 11 under diazepam and ketamine, and n = 9 under midazolam.

Distal and proximal microvascular perfusion in sedated animals (any treatment) was not different compared to that observed in control animals (distal perfusion: *P* = 0.11 (Fig 4A); proximal perfusion: *P* = 0.43 (Fig 4B), respectively). While slightly higher under alfaxalone, ketamine, and midazolam (318 (±170) PU; 351 (±183) PU; 294 (±202.0) PU, respectively), whereas both control and diazepam remained less so (166 (±62) PU; 185 (±124) PU, respectively). These differences were subject to a high degree of variability. Proximal perfusion, however, was much more consistent both within and between treatments. Proximal perfusion of control animals was 103±53 PU; however, outliers were observed in all treatments except midazolam. Temperature at probe sites did not differ between treatments (Distal: 33.2 (±0.5) °C; Proximal: 34.1 (±2) °C). Attrition of the sedated state meant that stable perfusion was obtained in 11/12 (92%) animals under diazepam and ketamine, and 9 (75%) under midazolam, whereas stable perfusion data was completed for the whole study in both alfaxalone and in control animals.

#### Respiration

**Fig 5:**
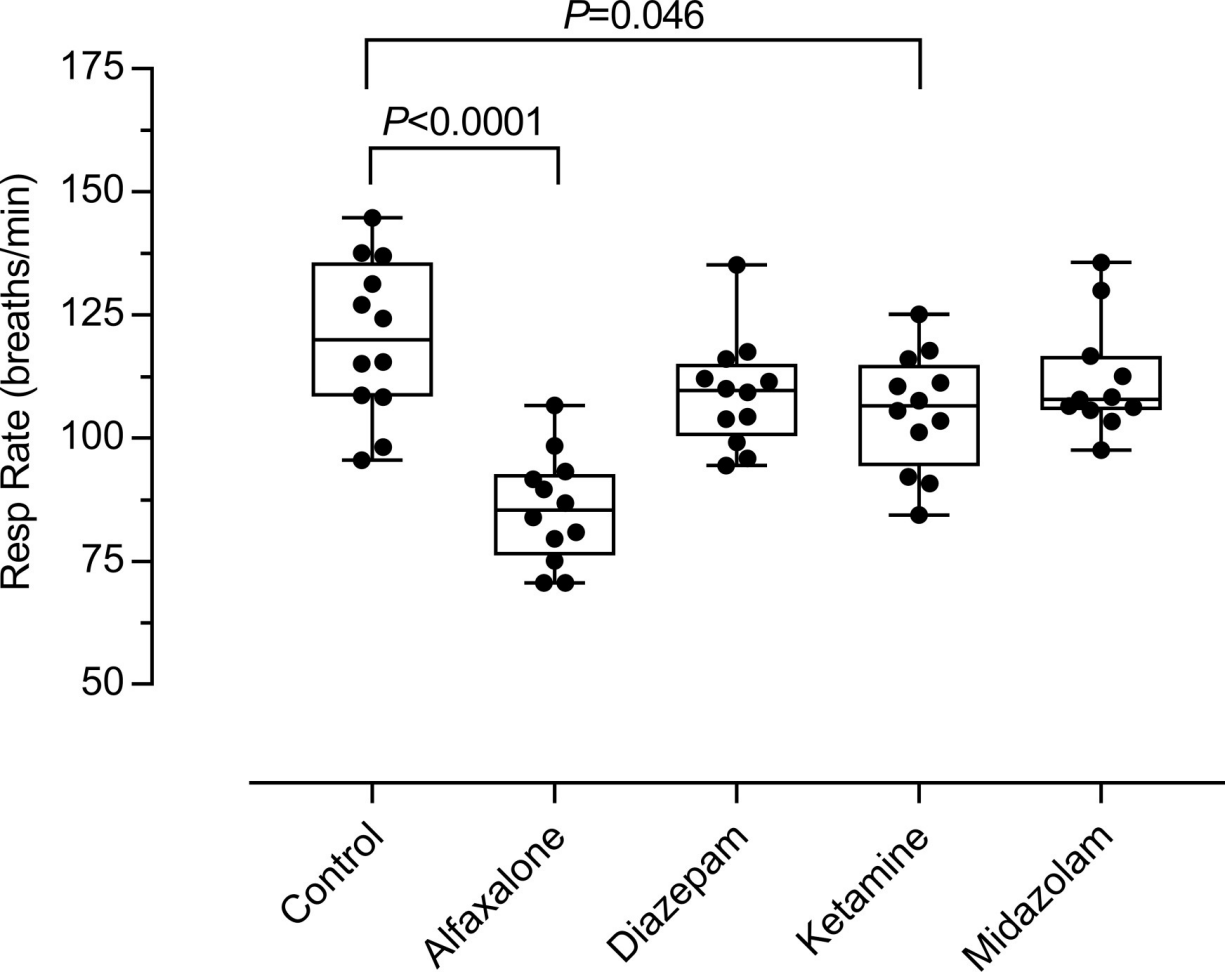
Respiratory response to different sedative agents. Significance indicates difference from control. Under midazolam n = 11.

Administration of sedatives reduced respiratory rate (RR) significantly below that of control animals (*P*<0.0001; Fig 5). RR in control animals was 120±10 breaths.min^-1^. Respiratory rate under both diazepam and midazolam was not significantly different from control (109±7 breaths.min^-1^ and 112±8 breaths.min^-1^, respectively). Whereas, under ketamine, respiration was reduced significantly to 106±8 breaths.min^-1^ (*P* = 0.05), with the greatest decrease in RR caused by alfaxalone (86±7 breaths.min^-1^; *P*<0.0001). Due to the instability of the sedated state under midazolam, data for 11 (92%) animals was suitable for analysis.

### Study II: Normative systolic blood pressure

Normative animals were similar in weight and age, to the animals involved in the sedation study (weight: 365.5±16.1g *P* = 0.69; age: 38±1d *P* = 0.87). Normative systolic blood pressure (SBP) was 60±4mmHg. When compared to the normative control group, all Study I groups displayed significantly higher SBP, including control (difference: 14.8mmHg, *P* = 0.0047). Alfaxalone was similarly different to the normative population as the control group (difference: 14.6; *P* = 0.0015), whereas ketamine produced the greatest difference in SBP (24.2mmHg, *P*<0.0001), with midazolam the second greatest difference (19.2mmHg, *P* = 0.0002), and diazepam presenting the least difference from the normative population (11.5mmHg, *P* = 0.014).

## Discussion

The current study has demonstrated that ketamine and aflaxalone are the most effective sedatives for providing stable light sedation for short duration cardiorespiratory monitoring (<1h) in guinea pigs. Further, at the dosage tested, midazolam was ineffective in sedating guinea pigs for physiological assessment. While all sedatives modified cardiorespiratory control, these variables remained within normal physiological limits, with ketamine exerting the least effect.

### Sedative efficacy

Sedation was most effectively achieved under ketamine. This was followed in efficacy by alfaxalone, with both agents producing rapid onset, reliable sedation and rapid recovery. In contrast, both midazolam and diazepam produced a much more volatile sedative state, characterised by hypersensitivity to manipulations (excessive motor response to light touch), and variable sedative efficacy, as well as varaible sedation onset. This was particularly apparent under midazolam to the extent that physiological characterisation was abandoned in a quarter of the animals. Hypersensitivty under midazolam has previously been observed cats and dogs [20], and has also been reported in response to alfaxalone administration in cats, dogs and humans [5, 21]. Of the agents currently tested, only ketamine has analgesic properties [6]. As such, the lack of analgesic properties in midazolam, diazepam and alfaxalone likely contributed to this agitation. Restraint was required when applying ECG electrodes under alfaxalone, midazolam, and diazepam, but once applied, animals under alfaxalone, and to a lesser extent diazepam, rapidly settled into moderate sedation.

For a single drug, sedative effects vary widely between species, with some agents proving excellent in some species and inadequate in others [2, 20]. This depends to a degree on route of adminstration and concentration of dose, but is highly dependent on metabolism and bioavailability. Guinea pigs frequently produce varied and contrary responses to sedative and anaesthetic agents [2]. For example, when administered medetomidine, an alpha-2 agonist and commonly used sedative, a recent study failed to achieve effective sedation in three out of four guinea pigs [2]. Similarly, when reviewing 10 years of experience Green et al. [22] noted that ketamine was an ineffective sedative in absence of adjuvants such as opioids or hypnotics. Although, as with the current study, sedation was still achieved with 40 mg.kg^-1^ despite intact pain responses. While sedative duration was not reported by Green [22], the current study observed ketamine sedation persisting for ~40 min. Green et al. [22] further noted, despite its shortcomings, that ketamine alone was still more effective than alfaxalone (alphaxalone-alphadolone-Cremophor-EL, old formulation [5]). When combined with xylazine, ketamine became a more stable sedative across species, and this combination remains a favoured sedative mix in guinea pigs, despite well recognised cardio-depressive effects [1, 22].

The efficacy of benzodiazepines also varies according to species and is further influenced by the use of adjuvant drugs [23]. In guinea pigs, sedation can be achieved using midazolam in combination with medetomidine and fentanyl (MMF) [3]. However, as with ketamine-xylazine (KZ), MMF produces marked cardiorespiratory depression [3]. When used alone, midazolam is unreliable in cats and dogs, producing paradoxical excitement and agression, but reliably induces sedation and muscle relaxation in swine, ferrets and birds [20]. The hypersensitivity to stimuli observed under midazolam in the current study appears to align with the findings in cats and dogs [21]. Interestingly, the current study did not observe hypersensitivity with diazepam, despite it belonging to the same family. As such, in guinea pigs ketamine and alfaxalone sedation proved most reliable.

### Physiological effects

While sedative depth is important for maintaining a stable compliant subject, the dosages and combinations of agents frequently come at the cost of cardiovascular and respiratory stability, as with KZ, and MMF [3]. The current study aimed to identify an effective agent for minimally invasive physiological monitoring that balanced these two needs. Indeed, none of the agents at the doses currently used produced dramatic changes in cardiovascular variables, and alfaxalone alone reduced respiration in a highly signifcant manner, albeit still well within normal ranges expected of guinea pigs [15].

All sedative agents currently investigated significantly increased HR. Diazepam and midazolam increased HR to a greater degree than alfaxalone and ketamine. Such effects have previously been ascribed to alterations in vascular tone [6, 24, 25], despite no differences observed in the cutaneous microvasculature nor blood pressure during the current study. Cardiovascular depression is commonly noted with most sedative agents including alfaxalone and diazepam or midazolam [26], with commonly used adjuvants potentiating their effects [27]. Ketamine acts directly and indirectly on the cardiovascular system to simultaneously depress and enhance function [6], such that its cardiovascular effects are minimal, and are only observed when an adjuvant exacerbates its efficacy (e.g., KZ [22]). Alfaxalone is increasingly used as a sedative as it also has limited cardiovascular effects [5, 24, 28–30]. However, alfaxalone does elicit dose-dependent respiratory depression [5, 30], as observed within the current study. Despite this, respiration remained within normal limits reported for guinea pigs (range: 40–130 breaths.min^-1^) [15]. The increased HR under alfaxalone was also observed in dogs (at 2 and 6 mg.kg^-1^), with Muir et al. [5] corroborating other findings [24, 25], suggesting this was due to decreased peripheral vascular resistance, secondary to vasodilation. Conversely, in a recent study of guinea pigs, d’Ovidio et al. [8] observed no cardiorespiratory effects of alfaxalone at the same dosage used currently. The benzodiazepines used in the current study had the most pronounced effects on HR. This is likely through a similar mechanism as Muir et al. [5] identified in alfaxalone, as benzodiazepines are known to produce vasodilation [6].

Resting normative blood pressure in the current study (Study II) was 60 mmHg, which is consistent with previous findings (~66 mmHg) [3]. However, when compared to the normative control group (Study II), SBP was higher in Study I control animals, likely due to a sequencing effect, whereby SBP was assessed following instrumentation of animals, whereas in Study II, animals had no injections or ECG or Doppler probe placement to confound readings. Systolic blood pressure in Study I remained unaffected by any of the currently used agents in comparison to control, despite any effects on HR and vasculature. As handling stress of the procedure was reduced, indicated by sedative depth, it is likely that a combination of a remainder of stress that the drugs did not remove, plus haemodynamic factors, such as peripheral dilation contributing to increased HR, maintained a slightly elevated SBP compared to the normative population. The greater variability in SBP observed during Study I reflects the inter-subject variability in sedative efficacy and stress of comprehensive physiological monitoring.

### Limitations

Diazepam and ketamine have recently been demonstrated to cause irritation and even tissue necrosis when administered SC or IM [9, 10]. Further, ketamine has been shown to be neurotoxic in both young and mature animals [7, 31], precluding their use as sedative agents [10]. The current study involved no formal assessments of behaviour, however, we observed no changes in animal behaviour (B.A.R. score) during the study period, suggesting no signs of neurodegeneration. This, however, does not discount that potential toxic effects would not have been observed histologically, with formal assessments, or after greater periods of time. Injection of diazepam and midazolam did result in acute irritation of the injection site, with animals often licking and biting at injection sites upon arousal, though this did not result in prolonged observable effects (all recovered by following day). As such, utilising ketamine or diazepam as an IM sedative agent for physiological assessments should only be performed in diluted, pH balanced quantities or in non-survival studies, to reduce the risk of their potential deleterious effects [10].

In contrast to ketamine, alfaxalone has been demonstrated to be safe in both young and mature animals [5, 8, 32, 33], despite GABAergic agents commonly being associated with neurotoxicity [34]. This is likely due to its neurosteroid properties [5, 8]. Additionally, alfaxalone has been shown to have no deleterious effects from repeat administration [35]. This makes alfaxalone more appropriate for studies requiring repeated physiological monitoring.

Alfaxalone, midazolam and diazepam have limited analgesic effects, and are therefore frequently paired with analgesic drugs. Indeed, in the current study, when applying the ECG needle electrodes, the painful stimulus may have led to increased arousal and a hyperresponsivity under all three agents. However, the purpose of the current study was to examine the capacity of the drug *per se* to facilitate physiological examination, and under situations where reducing the state of anxiety did not impair homeostatic functioning. Furthermore, the wide range of formulations (combination, or alone), differences in dosage and in the route of administration impair assessment of the efficacy of particular sedative agents. Adjuvants additionally interact to compound the sedated state, and depress cardiorespiratory function [1, 27, 36]. As such, additional agents were deemed to be a confounding influence and primarily deleterious in a study where analgesia was not required.

## Conclusions

Achieving a stable depth of sedation to enable detailed, physiologically robust, cardorespiratory characterisation remains challenging, especially in species like the guinea pig. Ketamine and alfaxalone produced the most stable sedation. However, the potential risks of neurotoxicity of ketamine and diazepam when administered IM may limit their use in repeated physiological monitoring. Furthermore, while benzodiazepines are effective as anxiolytics in some species, humans included, their sole use is not recommended in guinea pigs. Despite the limitations, most sedatives still enable more detailed physiological monitoring than can be reliably achieved in conscious animals. In conclusion, alfaxalone proved to be the most effective in juvenile guinea pigs and induced stable, short-acting sedation for physiological monitoring.

## Supporting information

S1 Table Monitored signs of movement and attrition rate during sedation with intramuscular sedatives. (DOCX)

S2 Table Monitored signs of body tone and attrition rate during sedation with intramuscular sedatives. (DOCX)

S3 Table Monitored signs of reaction to manipulation and attrition rate during sedation with intramuscular sedatives. (DOCX)

S4 Table Monitored signs of posture and attrition rate during sedation with intramuscular sedatives. (DOCX)

S5 Table Monitored signs of righting reflex and attrition rate during sedation with intramuscular sedatives. (DOCX)
