# Supplementary material for: Differential effects of four intramuscular sedatives on cardiorespiratory stability in juvenile guinea pigs (Cavia porcellus)
Source: PLoS One. 2021 Nov 15;16(11):e0259559. doi: 10.1371/journal.pone.0259559 (PMC8592412; doi:10.1371/journal.pone.0259559)
Supplement: S1 Table — (DOCX) [file pone.0259559.s001.docx]

**Supplemental Table 1.** Monitored signs of movement and attrition rate during sedation with intramuscular sedatives (Alfaxalone 5mg.kg^-1^, Diazepam 5mg.kg^-1^, Ketamine 30mg.kg^-1^, Midazolam 2mg.kg^-1^) in comparison to control (no sedative) in 12 guinea pigs undergoing physiological monitoring.

| Time Points | Movement | | | | |
| --- | --- | --- | --- | --- | --- |
|  | Control | Alfaxalone | Diazepam | Ketamine | Midazolam |
| 0 | 0±0 (12) | 0±0 (12) | 0±0 (11) | 0±0 (12) | 0±0 (12) |
| 5 | 1.7±0.7 (12) | 4.9±0.1 (12) | 3.5±0.6 (11) | 5.0±0.0 (12) | 2.7±0.5 (12) |
| 10 | 2.1±0.7 (12) | 4.4±0.2 (12) | 2.8±0.5 (11) | 4.4±0.3 (12) | 3.0±0.5 (12) |
| 15 | 2.1±0.7 (12) | 4.4±0.3 (11) | 3.7±0.4 (11) | 4.5±0.2 (12) | 3.0±0.4 (12) |
| 20 | 2.6±0.8 (11) | 3.4±0.5 (11) | 3.1±0.4 (11) | 4.3±0.3 (12) | 2.1±0.4 (12) |
| 25 | 3.2±0.7 (11) | 3.1±0.4 (12) | 3.4±0.5 (9) | 3.9±0.4 (12) | 2.3±0.4 (12) |
| 30 | 3.6±0.8 (8) | 2.2±0.6 (11) | 3.2±0.4 (11) | 4.1±0.5 (12) | 2.6±0.5 (11) |
| 35 | 1.0±0.8 (6) | 1.4±0.9 (5) | 2.5±0.5 (10) | 4.1±0.5 (11) | 1.5±0.8 (6) |
| 40 |  |  | 3.3±0.6 (9) | 3.7±0.5 (11) | 2.5±2.5 (2) |
| 45 |  |  | 2.8±0.7 (8) | 3.1±0.6 (9) | 2.5±2.5 (2) |
| 50 |  |  | 1.8±0.7 (6) | 2.0±1.0 (3) | 5.0±0.0 (1) |
| 55 |  |  | 1.0±0.0 (1) | 1.5±1.5 (2) |  |
| 60 |  |  |  | 2.0±0.0 (1) |  |

N.b. Data are reported as mean±SEM (n)
