# Supplementary material for: Differential effects of four intramuscular sedatives on cardiorespiratory stability in juvenile guinea pigs (Cavia porcellus)
Source: PLoS One. 2021 Nov 15;16(11):e0259559. doi: 10.1371/journal.pone.0259559 (PMC8592412; doi:10.1371/journal.pone.0259559)
Supplement: S3 Table — (DOCX) [file pone.0259559.s003.docx]

**Supplemental Table 3.** Monitored signs of reaction to manipulation and attrition rate during sedation with intramuscular sedatives (Alfaxalone 5mg.kg^-1^, Diazepam 5mg.kg^-1^, Ketamine 30mg.kg^-1^, Midazolam 2mg.kg^-1^) in comparison to control (no sedative) in 12 guinea pigs undergoing physiological monitoring.

| Time Points | Reaction to Manipulation | | | | |
| --- | --- | --- | --- | --- | --- |
|  | Control | Alfaxalone | Diazepam | Ketamine | Midazolam |
| 0 | 0±0 (12) | 0±0 (12) | 0±0 (12) | 0±0 (12) | 0±0 (12) |
| 5 | 0.1±0.1 (12) | 2.4±0.1 (12) | 1.1±0.2 (12) | 2.8±0.1 (12) | 1.6±0.2 (12) |
| 10 | 0.4±0.2 (12) | 1.5±0.3 (11) | 1.7±0.2 (12) | 2.5±0.2 (11) | 1.3±0.3 (12) |
| 15 | 0.5±0.2 (11) | 1.6±0.4 (12) | 1.5±0.3 (11) | 2.1±0.3 (12) | 1.2±0.5 (12) |
| 20 | 0.7±0.4 (12) | 1.8±0.4 (12) | 0.8±0.4 (12) | 2.0±0.3 (12) | 1.1±0.4 (12) |
| 25 | 0.8±0.3 (12) | 1.2±0.3 (12) | 1.5±0.4 (12) | 2.0±0.3 (12) | 0.8±0.2 (12) |
| 30 | 1.1±0.4 (8) | 0.5±0.2 (11) | 1.4±0.3 (12) | 2.1±0.3 (12) | 0.9±0.3 (11) |
| 35 | 0.5±0.3 (6) | 0.2±0.2 (5) | 1.0±0.2 (12) | 1.8±0.2 (11) | 1.0±0.4 (6) |
| 40 | 0±0 (2) |  | 1.1±0.4 (9) | 1.1±0.3 (11) | 0.5±0.5 (2) |
| 45 |  |  | 1.0±0.3 (8) | 0.9±0.4 (8) | 0.5±0.5 (2) |
| 50 |  |  | 1.2±0.4 (6) | 1.0±0.6 (3) | 0±0(1) |
| 55 |  |  |  | 0.5±0.5 (2) |  |
| 60 |  |  |  | 1.0±0.0 (1) |  |

N.b. Data are reported as mean±SEM (n)
